# Bowel pseudo-obstruction caused by goblet cell adenocarcinoma of the appendix: A case report

**DOI:** 10.1016/j.ijscr.2024.109938

**Published:** 2024-06-22

**Authors:** Akifumi Okada, Shoichiro Mukai, Yasufumi Saito, Toshihiro Nishida, Toshikatsu Fukuda, Hideki Ohdan

**Affiliations:** a Department of Surgery, Chugokurosai Hospital, 1-5-1, Hirotagaya, Kure City, Hiroshima 737-0193, Japan; b Department of Pathology, Chugokurosai Hospital, 1-5-1, Hirotagaya, Kure City, Hiroshima 737-0193, Japan; c Department of Gastroenterological and Transplant Surgery, Graduate School of Biomedical and Health Sciences, Hiroshima University, 1-2-3 Kasumi, Minami-ku, Hiroshima 734-8551, Japan

**Keywords:** Goblet cell adenocarcinoma of appendix, Bowel pseudo-obstruction, Auerbach's plexus, Case report

## Abstract

**Introduction:**

Goblet cell adenocarcinoma of the appendix is a rare diagnosis with features of both adenocarcinomas and carcinoid tumors. Commonly presenting with chronic abdominal pain, appendicitis, or abdominal distention, it can also be incidentally discovered during appendectomies.

**Case presentation:**

A 50-year-old man with right lower abdominal pain was admitted to our hospital, which is a critical care center. A computed tomography(CT) scan showed ileal narrowing, but endoscopy found no strictures. He was admitted with suspected bowel obstruction and improved with an ileal tube. Laparoscopic surgery revealed a tumor of the appendix. Histologically, he was diagnosed goblet cell adenocarcinoma, suggesting tumor infiltration of nerve fibers impairing peristalsis.

**Discussion:**

Goblet cell adenocarcinoma of the appendix has unique histology and a poor prognosis. Treatment typically involves surgery and chemotherapy. This case highlights challenges in preoperative diagnosis, with the tumor causing bowel pseudo-obstruction by invading the intestinal wall and nerve plexus. Extensive infiltration of Auerbach's plexus was observed, consistent with the length of intestinal stenosis.

**Conclusion:**

This case describes goblet cell adenocarcinoma of the appendix leading to bowel pseudo-obstruction due to ileal end stenosis. It emphasizes the importance of considering this diagnosis in cases of bowel obstruction without an obvious mass.

## Highlights

• Goblet cell adenocarcinoma of appendix is a rare diagnosis and associated with a poor prognosis.
• Surgery is the most common treatment of goblet cell adenocarcinoma of appendix.
• Goblet cell adenocarcinoma of appendix is a rare cause of bowel pseudo-obstruction.

## 1 Introduction

Goblet cell adenocarcinoma of the appendix (alternatively called goblet cell carcinoid [GCC]) is a rare diagnosis, with an age-adjusted incidence of 0.5/1,000,000 per year [1]. Goblet cell adenocarcinoma is characteristically biphasic and derived from pluripotent intestinal stem cells that differentiate into mucinous and neuroendocrine cells [2]. Thus, it shares histological features with both adenocarcinomas and carcinoid tumors, respectively.

The most common presentations are chronic abdominal pain, appendicitis, and abdominal distention; however, these are also frequently found incidentally during appendectomies [3].

Here, we describe a rare case in which a patient developed a bowel pseudo-obstruction caused by appendiceal goblet cell adenocarcinoma.

## 2 Case presentation

**Fig. 1:**
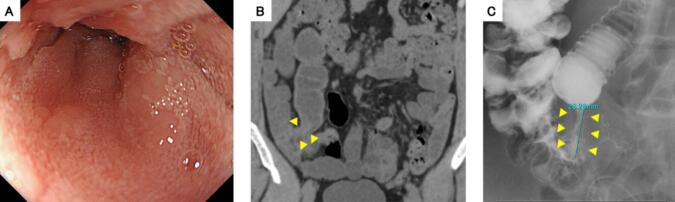
Arrowhead: stenosis of the terminal ileum. A: Lower gastrointedtinal endoscopy showed no abnormalities in the intestinal mucosa. B: Abdominal CT revealed narrowing of the terminal ileum and dilation of the oral intestinal lumen. C: Ileus tube angiography confirmed the stenosis of the terminal ileum.

**Fig. 2:**
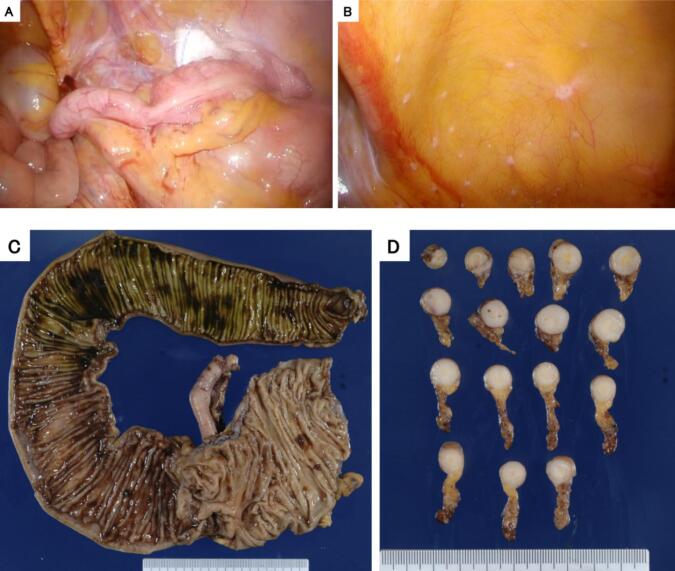
A: Intraoperative findings indicated moderate swelling of the appendix. B: Multiple disseminated nodules were observed on the peritoneum and the intestine surface. C: No organic stenosis that could have led to intestinal obstruction was identified. D: The short axial sectioned plane image of the appendix revealed complete destruction of appendiceal structures by the tumor.

A 50-year-old man with a one-month history of right-sided abdominal pain was presented to our hospital by walk-in. On arrival at our hospital, patient's vital signs stabilized. His abdomen was unremarkable, and intestinal peristalsis was normal. The patient had no relevant medical history. Trimebutine maleate and lafutidine were then administered. He drank 350 mL of beer every day. He had smoked 20 cigarettes a day for 20 years and quit smoking when he was aged 40 years. Laboratory studies were unremarkable. CEA and CA19-9 were within normal range. Abdominal CT revealed narrowing of the terminal ileum and dilation of the more proximal part of the intestine lumen (Fig. 1B). Lower gastrointestinal endoscopy and biopsy revealed that there were no specifical findings on the intestinal mucosa, edema or intestinal lumen narrowing (Fig. 1A). Bowel obstruction was suspected, and a 190 cm ileal tube was inserted to the small intestine. Ileus tube contrast revealed terminal ileal stenosis and this stenosis did not improve after four days (Fig. 1C). Because bowel pseudo-obstruction did not improve with conservative treatment and the patient could not eat, the patient underwent surgery on the 13th day to improve symptoms and investigate the cause of the pseudo-obstruction. The surgeon had experience performing >100 laparoscopic ileocecal resection. Intraoperative findings showed that the appendix was swollen, but no tumor caused narrowing of the intestinal lumen at the end of the ileum (Fig. 2A). There were multiple nodules on the peritoneal and intestinal surface (Fig. 2B). Frozen section from peritoneal surface diagnosis during surgery suggested metastatic carcinoma. A laparoscopic ileocecal resection with D3 lymphadenectomy was performed. Anastomosis was done by instrumented anastomosis, using linear stapler. Surgery time was 241 min. Postoperative course was good, and the patient was discharged 14 days postoperatively. Length of intensive care unit stay was 0 day.

**Fig. 3:**
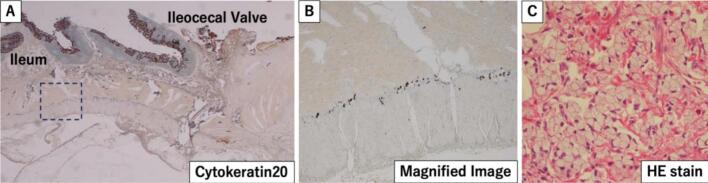
Histological findings. A: The tumor invades along the Auerbach's plexus of the ileum. B: The tumor cells are stained. C: HE staining: the tumor cells, like goblet cells, were arranged in glandular ducts.

**Fig. 4:**
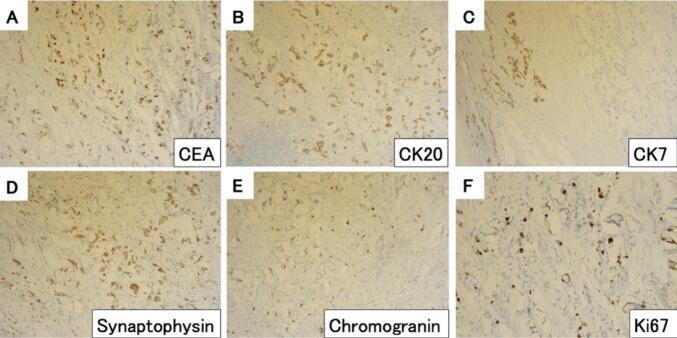
Immunostaining was positive for CEA and CK20, and partially positive for CK7. Synaptophysin and Chromogranin were positive, and Ki67 positivity was approximately 20 %–50 %.

Histopathological examination of the specimen revealed that the appendix was grayish-white and elastic-hard, the wall was diffusely thickened, the lumen was obliterated, and the appendiceal structures were completely destroyed by the tumor (Fig. 2C, D). Histologically, tumor cells such as goblet cells were arranged in glandular ducts, in areas of poorly differentiated mucinous carcinoma floating in the mucus, or in areas where ring cells grew in full-blown foci (Fig. 3C). The tumor cells infiltrate the wall and grow invasively within the nerve fibers or vessel walls of the mesoappendix. The tumor cells spread from the wall of the appendix, infiltrated into the cecum and ileum walls around the orifice of the vermiform appendix, and extensively infiltrated into the surrounding area along Auerbach's plexus (Fig. 3A, B). The tumor cells had abundant mucus with positive Periodic acid Schiff (PAS) staining, positive for CK20, CK7 in some cases, chromogranin, and synaptophysin, with Ki67 positivity ranging 20 %–50 % (Fig. 4). The tumor was diagnosed as a goblet cell adenocarcinoma because it was a poorly differentiated mucinous adenocarcinoma with endocrine differentiation, histologically an adenoendocrine carcinoma showing a goblet cell-like form, and was the primary tumor of the appendix. The tumor had invaded the serosal surface, and the dissected lymph nodes showed only a few metastases in the regional lymph nodes; however, disseminated nodules were observed on the serosal surface of the ileum and Douglas fossa. Therefore, the goblet cell adenocarcinoma of the appendix was diagnosed as T4aN1bM1b Stage IVB (TNM Classification of Malignant Tumors, 8th Edition). Mild luminal dilatation at the ileum end, mild stenosis mainly at the ileocecal valve, and wall induration were observed. However, the mucosal folds were preserved and soft, and no stenosis that could have led to bowel obstruction was identified.

Five courses of chemotherapy with FOLFOXIRI were administered, and once chemotherapy was completed, the patient was followed up. Two years and three months later, CT scan revealed bowel obstruction resulting from recurrent peritoneal dissemination. After improvement of bowel obstruction, five courses of chemotherapy with CAPOX + BEV were administered. Three years and three months after the initial surgery, the patient died because of repeated bowel obstruction caused by multiple peritoneal disseminations.

## 3 Discussion

GCC of the appendix is classified as a subtype of adenocarcinoma. This classification is attributed to its distinct histology, which differs from carcinoid tumors composed solely of endocrine cells. GCC is associated with a poor prognosis, mainly due to peritoneal dissemination and metastasis [4]. Cases occurring due to bowel obstruction caused by peritoneal dissemination and cases in which the enlarged appendix adhered to the intestinal tract and formed a band have been reported [5,6]. However, no report have clearly stated the absence of organic stenoses.

Surgery is the most common treatment; however, no techniques have been established. Appendectomy or right hemicolectomy with lymph node dissection is often performed [2]. In patients with T3 and T4 tumors, there was a trend towards improved survival with right hemicolectomy, with no difference in survival seen in T1 and T2 tumors, regardless of appendicectomy or hemicolectomy [7].

For chemotherapy, a 5-FU–based regimen similar to that used to treat cancer is recommended [2]. Adenocarcinoma cells grow invasively in small luminal, lobular, and cord-like aggregates of varying sizes and shapes, as well as in individual cells in deep mucosal and submucosal layers with indistinct boundaries. Vascular invasion and nerve involvement are frequently observed. Mucous cells have mucous stains (PAS stain, Alcian blue stain), positive mucous vacuoles, and are positive for the mucin core protein, MUC2. Cancer cells that differentiate into endocrine cells are positive for the endocrine markers, chromogranin, and synaptophysin. Approximately half of cancer cells are positive for CEA, highly positive for cytokeratin 20 (CK20), and positive for CK7 [8,9].

Tang et al. classified the cases into three groups: group A, characterized by the neoplastic growth of normal goblet cells; group B, where annulus-like cells predominate; and group C, where areas indistinguishable from poorly differentiated adenocarcinoma are present [10]. The 5-year survival rates for disseminated cases were 100 % in group A, 38 % in group B, and 0 % in group C, indicating a poor prognosis. Tang classification correlated with survival: the median OS was 118 months in Tang group A, 83 months in Tang group B, and 20 months in Tang group C. The Ki-67 index, which is used to assess the malignancy of neuroendocrine tumors, showed no clear association with prognosis [9]. Certain findings in this case resembled those of poorly differentiated mucinous adenocarcinomas and were classified as group C.

In this case, CT, endoscopy, and blood tests showed no notable findings, and no apparent cause of bowel obstruction was identified preoperatively. Pathological examination revealed primary goblet cell adenocarcinoma of the appendix, which was thought to be a peristaltic disorder due to tumor invasion of the intestinal wall and severe invasion of the nerve plexus. The tumor cells spread from muscular layer to the subserosal membrane of cecum and ileum and were not exposed to the mucosal or serosa membrane surface, so we consider the gross appearance of these areas was normal. Achalasia and Hirschsprung's disease are known to cause bowel pseudo-obstruction without organic stenosis. Abnormalities in the muscular interlaminar plexus of the intestinal tract may be associated with bowel pseudo- obstruction in both diseases [11,12].

There was extensive (>2 cm) infiltration of the Auerbach's plexus from the end of the ileum to the mouth, which is generally consistent with the length of intestinal stenosis (28 mm) on ileal tube angiography.

## 4 Conclusion

Here, we report a case of goblet cell adenocarcinoma of the appendix that developed due to bowel pseudo-obstruction caused by ileal end stenosis. In cases of bowel obstruction due to stenosis of the ileal end without an obvious mass, goblet cell adenocarcinoma of the appendix should be considered in the differential diagnosis.

This work has been reported in line with the SCARE 2023 criteria [13].

## Consent

Written informed consent was obtained from the patient for publication of this case report and accompanying images. A copy of the written consent is available for review by the Editor-in-Chief of this journal on request.

## Ethical approval

A case report is exempt from ethical approval in our institution.

## Funding

There are no sources of funding to declare.

## Author contribution

AO drafted the article. SM had revised the manuscript critically. YS was assistants of operation. TN revised the histopathological findings. HO and TF were supervision of this manuscript. All authors contributed to study concept or design at this submission and approved the final version.

## Guarantor

Dr. Shoichiro Mukai.

## Registration of research studies

Not applicable.

## Declaration of competing interest

The authors declared no conflict of interest.
